# The Influence of Air Pressure in Electric Arc Spraying on the Properties of 30KhGSA/20Kh13 Bilayer Coatings

**DOI:** 10.3390/ma19020232

**Published:** 2026-01-07

**Authors:** Dastan Buitkenov, Aiym Nabioldina, Aibek Alibekov, Yermakhan Molbossynov

**Affiliations:** 1 Research Center Surface Engineering and Tribology, Sarsen Amanzholov East Kazakhstan University, Ust-Kamenogorsk 070000, Kazakhstan; dbuitkenov@vku.edu.kz (D.B.); aibek.alibekov@mail.ru (A.A.); 2 Protective and Functional Coatings Scientific Center, Daulet Seikbayev East Kazakhstan Technical University, Ust-Kamenogorsk 070010, Kazakhstan; mr.molbosunov@gmail.com

**Keywords:** electric arc spraying, wear resistance, 20Kh13, 30KhGSA, microhardness, elastic modulus, tribology, coefficient of friction

## Abstract

This study investigates the structural formation and performance characteristics of coatings produced by electric arc spraying using 20Kh13 steel wire on a 45 steel substrate with an adhesive interlayer made of 30KhGSA steel. Particular attention is paid to the effect of spraying air pressure (0.4–0.6 MPa) on the morphology, phase composition, microhardness, elastic modulus, and tribological properties of the resulting coatings. Microstructural and X-ray phase analyses revealed that increasing air pressure leads to higher coating density, reduced porosity (~2–3%), and increased content of the Fe_3_O_4_ oxide phase. Nanoindentation tests showed that the highest microhardness (up to 270 HV) and elastic modulus values were observed at 0.4 MPa, while the greatest structural integrity and stable frictional behavior were achieved at 0.6 MPa. Under both dry and lubricated conditions, the coatings exhibited stable performance and a low coefficient of friction (0.10–0.12 in oil), confirming the potential of the developed technology for the restoration and strengthening of component surfaces operating under combined loading and aggressive environmental conditions.

## 1. Introduction

The working surfaces of shafts, axles, and transmission elements of rotating equipment are among the most vulnerable components of machines. Their operation under combined loads—including contact friction, vibrations, temperature fluctuations, impact forces, and aggressive environmental factors (humidity, dust, chemical reagents)—leads to the development of various types of wear: abrasive, adhesive, fatigue, and corrosion-mechanical wear [1,2,3,4,5,6]. As a result, surface layer degradation, changes in the geometry of mating parts, increased clearances, higher roughness, and reduced motion transmission accuracy are observed. All of these factors lead to decreased equipment reliability and increased operating costs. The wear problem is especially critical at heavy engineering enterprises, where equipment operates under high loads and in aggressive environments [7,8,9]. According to modern studies [10], up to 60% of failures in rotating units are associated with combined corrosion–tribotechnical wear. This drives a high demand for effective technologies for restoring and protecting working surfaces that can ensure reliable operation of equipment under extreme conditions. Despite the availability of various strengthening methods, many of them either do not provide the required level of adhesion and wear resistance or are associated with high cost and implementation complexity. Against this background, the task of extending service life and restoring components using surface modification technologies, particularly thermal spraying, is of current relevance [11,12,13,14,15].

Among the various thermal strengthening methods, arc metallization (AM) stands out for its technological simplicity, equipment availability, and high efficiency under harsh operating conditions [16,17,18]. AM enables the production of dense, wear-resistant, and corrosion-resistant coatings with good adhesion and minimal thermal impact on the base material, which is critical when restoring mating and fitted surfaces. According to recent studies, the use of AM can significantly increase the service life of components subjected to intense friction and exposure to aggressive environments [19,20,21,22,23]. In particular, it has been established that coatings produced by arc spraying using stainless steel wires exhibit improved characteristics compared to electroplated and some plasma coatings [24]. This opens up opportunities for selecting optimal wire materials, taking into account the specific operating conditions of the restored surfaces.

In the present study, a wire based on 20Kh13 steel, known for its enhanced corrosion resistance, was selected for the top coating layer, while 30KhGSA structural steel was used as the interlayer to ensure strong adhesion to the substrate and resistance to mechanical loads. The combined “interlayer–coating” system is effective for restoring shafts and axles operating under conditions of vibration, variable loads, and contact with aggressive technological environments. However, it should be noted that the influence of technological parameters, particularly the spraying air pressure, on the properties of 20Kh13 coatings produced by electric arc spraying has not been sufficiently studied to date. Meanwhile, this parameter has a significant effect on particle kinetic energy, deposition quality, oxidation degree, and consequently, on the structure and performance characteristics of the coatings.

In this regard, the aim of the present work is to investigate the structure and performance properties of coatings obtained by electric arc spraying using 20Kh13 steel wire and a 30KhGSA interlayer, at various air pressure values. Special attention is given to the assessment of microhardness and tribological characteristics of the coatings under dry friction and lubricated conditions.

## 2. Materials and Methods

Electric arc spraying was carried out using an SX600-type unit (Guangzhou Sanxin Metal S&T Co., Ltd., Guangzhou, Guangdong, China) designed for the deposition of metallic coatings by the wire arc spraying technique. In this process, cored metallic wires are used as the feedstock; the wire tips are instantaneously melted by an electric arc and atomized by a compressed gas stream into fine molten droplets, which are then propelled toward and deposited onto the substrate surface.

In the present study, cored wires with a diameter of 1.6 mm were employed. A 30KhGSA alloy wire was used for the formation of the interlayer, while a 20Kh13 alloy wire was used to produce the main functional coating layer. The 30KhGSA alloy steel is a low-alloy structural steel containing approximately 0.28–0.34 wt.% C, 0.8–1.1 wt.% Cr, 0.8–1.1 wt.% Mn, and 0.9–1.2 wt.% Si, which provides good strength and adhesion when used as an interlayer material. The 20Kh13 steel is a martensitic stainless steel with a typical composition of 0.16–0.25 wt.% C and 12–14 wt.% Cr, offering enhanced corrosion and wear resistance and therefore serving as the main functional coating layer. The coatings were deposited onto substrates made of 45 structural steel. According to ASTM A108 [25], steel 45 has the chemical composition listed in Table 1.

Surface preparation included sandblasting using electrocorundum, removal of abrasive residues with compressed air, followed by cleaning in an ultrasonic bath with ethanol. After treatment, the samples were dried in a drying oven at a temperature of 60 °C until complete removal of moisture. The substrates were then fixed in a holder and heated to 200–250 °C, which contributed to improved coating adhesion.

A cored wire made of 30KhGSA steel was used as the interlayer, applied under standard parameters: current—130 A, voltage—60 V, air pressure—0.6 MPa, distance to the surface—20 cm, spraying time—5 s. The top layer was applied using a cored wire of 20Kh13 steel, with the main variable parameter being the pressure of the supplied compressed air. The values of other parameters (voltage, current, distance to the surface, spraying time) remained constant. The spraying modes are shown in Table 2.

X-ray phase analysis (XRD) of the coatings was carried out using a PANalytical X’PERT PRO diffractometer (PANalytical, Almelo, The Netherlands) with CuKα radiation. The coating morphology was examined using a scanning electron microscope SEM 3200 (CIQTEK Co., Ltd., Hefei, China), equipped with an energy-dispersive X-ray spectroscopy (EDS) system XFlash Detector 730M-300 (Bruker Nano GmbH, Berlin, Germany). The microhardness of the obtained layers was measured in accordance with the DIN EN ISO 14577-1 standard [26] using a FISCHERSCOPE^®^ HM2000 S microhardness tester (Helmut Fischer GmbH, Sindelfingen, Germany) equipped with a Vickers diamond indenter. The applied maximum load was 2000 mN. The loading and unloading durations were 20 s each, with a dwell time of 5 s at the maximum load, resulting in a total indentation cycle time of 45 s. For each sample, at least 10 indentations were performed at different locations, and the reported microhardness values represent the average with the corresponding standard deviation.

**Figure 1:**
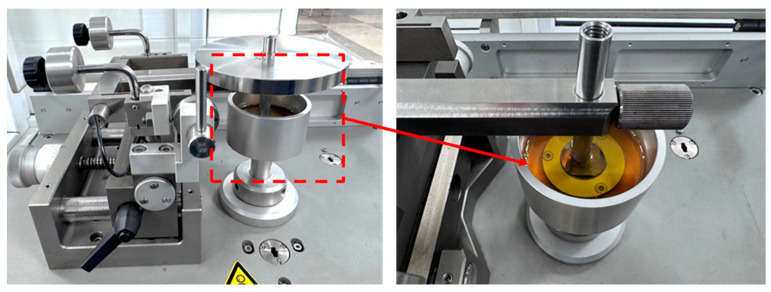
General view of the TRB3 tribometer for conducting tribological tests.

Tribological tests of the electric arc spraying coatings were carried out on a TRB3 tribometer (Anton Paar, Graz, Austria) using a ball-on-disk configuration. A 6 mm diameter chromium steel ball was used as the counterbody. The tests were conducted under a load of 10 N, a sliding speed of 5 cm/s, and a total sliding distance of 200 m. The study was performed in two modes: under dry friction conditions and using oil as a lubricating medium (Figure 1).

## 3. Results

**Figure 2:**
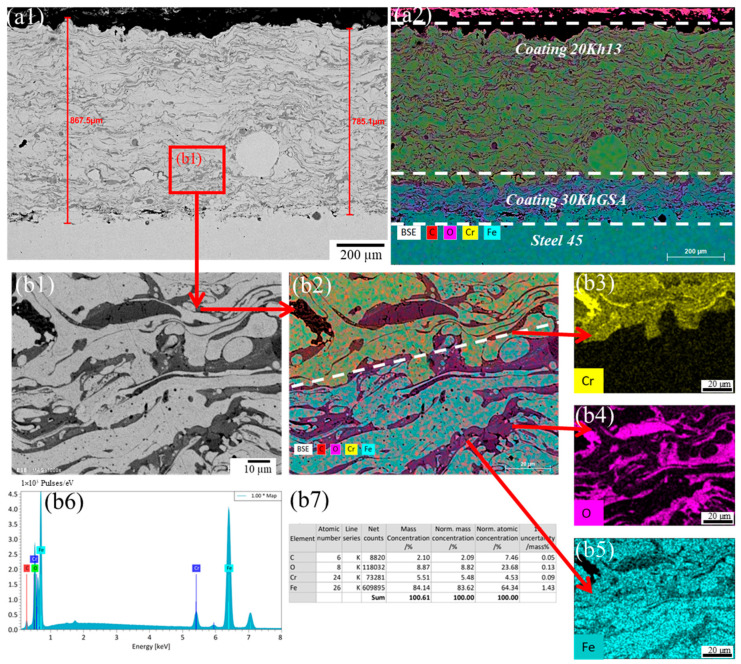
Microstructure of the coating deposited at an air pressure of 0.4 MPa: (**a**) cross-sectional SEM image of the multilayer coating deposited on steel 45, showing the total coating thickness; (**a1**) backscattered electron (BSE) image combined with EDS elemental mapping of the coating cross section, indicating the 20Kh13 top layer, 30KhGSA interlayer, and steel 45 substrate; (**a2**) corresponding EDS elemental overlay map of the same region; (**b1**) high-magnification SEM image of the selected region within the coating, illustrating the lamellar microstructure; (**b2**) corresponding EDS elemental overlay map of the same region; (**b3**–**b5**) EDS mapping of chromium (Cr), oxygen (O), and iron (Fe); (**b6**) EDS spectrum acquired from the selected area; (**b7**) quantitative EDS elemental composition of the analyzed region.

**Figure 3:**
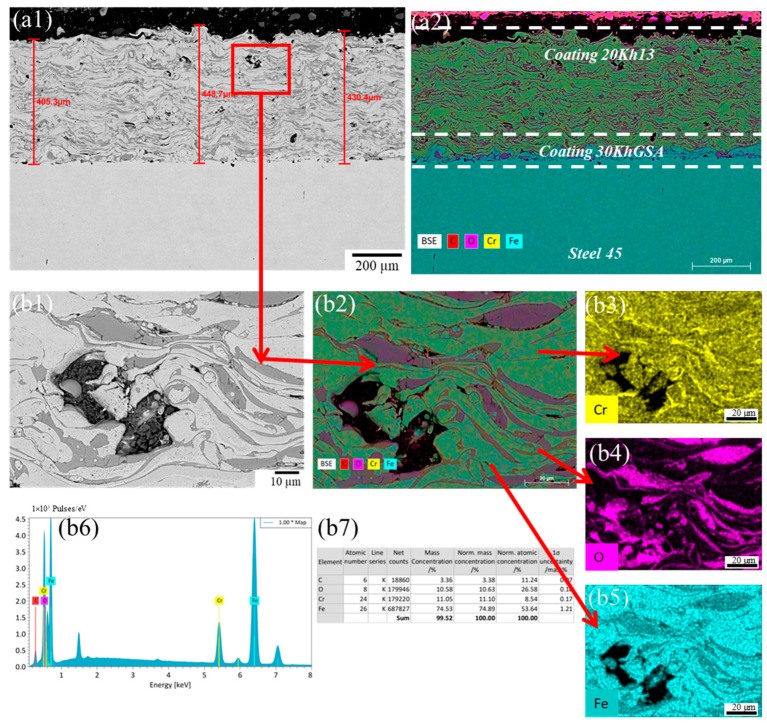
Microstructure of the coating deposited at an air pressure of 0.5 MPa: (**a**) cross-sectional SEM image of the multilayer coating deposited on steel 45, showing the total coating thickness; (**a1**) backscattered electron (BSE) image combined with EDS elemental mapping of the coating cross section, indicating the 20Kh13 top layer, 30KhGSA interlayer, and steel 45 substrate; (**a2**) corresponding EDS elemental overlay map of the same region; (**b1**) high-magnification SEM image of the selected region within the coating, illustrating the lamellar microstructure; (**b2**) corresponding EDS elemental overlay map of the same region; (**b3**–**b5**) EDS mapping of chromium (Cr), oxygen (O), and iron (Fe); (**b6**) EDS spectrum acquired from the selected area; (**b7**) quantitative EDS elemental composition of the analyzed region.

**Figure 4:**
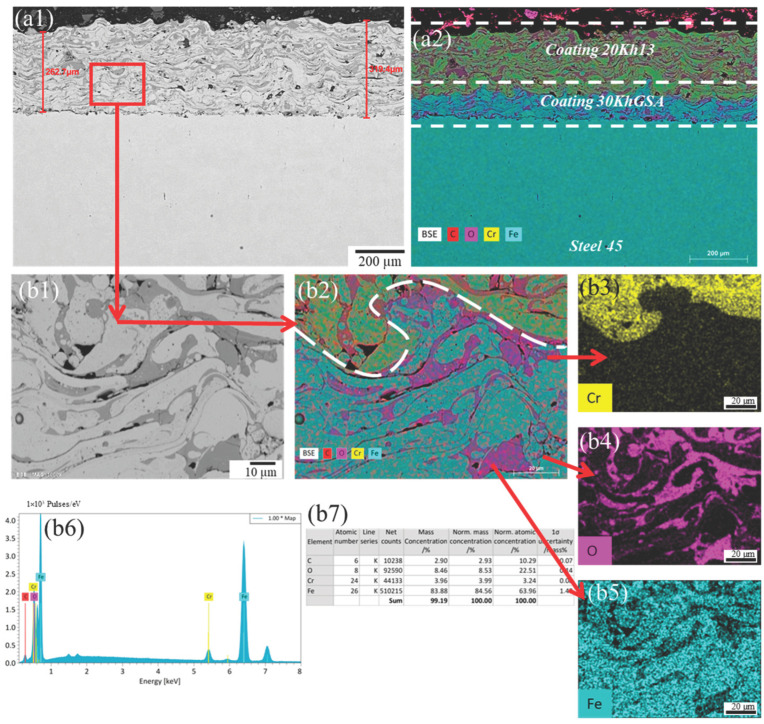
Microstructure of the coating deposited at an air pressure of 0.6 MPa: (**a**) cross-sectional SEM image of the multilayer coating deposited on steel 45, showing the total coating thickness; (**a1**) backscattered electron (BSE) image combined with EDS elemental mapping of the coating cross section, indicating the 20Kh13 top layer, 30KhGSA interlayer, and steel 45 substrate; (**a2**) corresponding EDS elemental overlay map of the same region; (**b1**) high-magnification SEM image of the selected region within the coating, illustrating the lamellar microstructure; (**b2**) corresponding EDS elemental overlay map of the same region; (**b3**–**b5**) EDS mapping of chromium (Cr), oxygen (O), and iron (Fe); (**b6**) EDS spectrum acquired from the selected area; (**b7**) quantitative EDS elemental composition of the analyzed region.

Microstructural analysis of the coatings obtained by electric arc spraying using a two-layer scheme (30KhGSA interlayer and 20Kh13 top layer) revealed characteristic features of the material structure formation. In the cross section of the coatings, three zones are clearly distinguishable: the substrate (steel 45), the interlayer, and the top layer (Figure 2, Figure 3 and Figure 4). The interlayer performs an adhesion function, ensuring reliable bonding between the sprayed metal and the substrate, as well as helping to reduce thermal stresses during the application of the top layer.

The top layer formed from 20Kh13 steel exhibits the typical layered structure characteristic of arc spraying, which is due to the sequential deposition and solidification of particles. The micrographs show alternating wavy layers corresponding to solidified metal layers and oxide inclusions. The latter are formed as a result of partial oxidation of particles during their flight through the air. In addition, the coating contains pores of various shapes: discoidal (likely arising between layers due to insufficient wetting) and globular (associated with incomplete filling during particle solidification). Also observed are individual unmelted rounded particles, indicating uneven wire feed or local temperature fluctuations. Additionally, during elemental distribution analysis (EDS-mapping), it was found that at the boundary between the 30KhGSA interlayer and the 20Kh13 top coating there is a decrease in chromium concentration (Figure 2(a2), Figure 3(a2) and Figure 4(a2)). This is explained by the difference in the chemical compositions of the alloys used (in 20Kh13, Cr content is about 13%, whereas in 30KhGSA it does not exceed 1%).

To evaluate the influence of process parameters on the coating morphology, an analysis of samples obtained at different air spraying pressures—0.4, 0.5, and 0.6 MPa—was conducted. This made it possible to identify patterns reflecting the relationship between the kinetic energy of the particles, the degree of their deposition, and the quality of the formed layer.

Thus, at the minimum air pressure of 0.4 MPa (sample No. 1), the greatest coating thickness was recorded—up to 821.1 ± 41.1 µm. This is explained by the reduced particle velocity, which facilitates their deposition and accumulation on the surface. However, the coating structure also showed interparticle voids and unmelted inclusions, indicating its heterogeneity and insufficient density (Figure 2). Image analysis of SEM cross sections revealed an approximate porosity of ~6% for this coating.

With an increase in pressure to 0.5 MPa (sample No. 2), the coating thickness decreases to 448.4 ± 36.9 µm; however, the layer structure becomes more uniform. A reduction in the number of defects and improved particle cohesion indicate improved coating quality due to an optimal balance between particle velocity and the amount of deposited material (Figure 3). The porosity in this case decreases to approximately 3%.

The highest structural density was demonstrated by sample No. 3, obtained at the maximum air pressure of 0.6 MPa. In this case, the coating thickness was 315.7 ± 15.8 µm, which is attributed to the intense material loss due to the high-speed air jet. However, such dynamics contribute to better compaction of the coating and a reduction in the number of pores (Figure 4), despite the decrease in layer thickness. The porosity of this coating was estimated to be below ~2–3%, despite the reduced thickness.

Microstructural analysis of the coatings obtained by electric arc spraying showed that the formation of the coating structure occurs under extremely high cooling rates. This is due to the fact that the molten metal particles are small in size and strike the surface at high speed, solidifying almost instantaneously. Such conditions of coating formation directly affect its phase composition, which makes it justified to move from morphological analysis to a detailed study of phase formation. As the microstructural investigation revealed, the coating contains alternating metallic layers, oxide interlayers, individual rounded particles, as well as pores of various shapes. The presence of oxides and the inhomogeneity of the layered structure indicate intensive oxidation of particles during the spraying process. These observations allowed the hypothesis that changes in spraying parameters—in particular, air pressure—would directly affect the phase composition of the coating. To test this hypothesis, a phase analysis of the sprayed samples was carried out.

**Figure 5:**
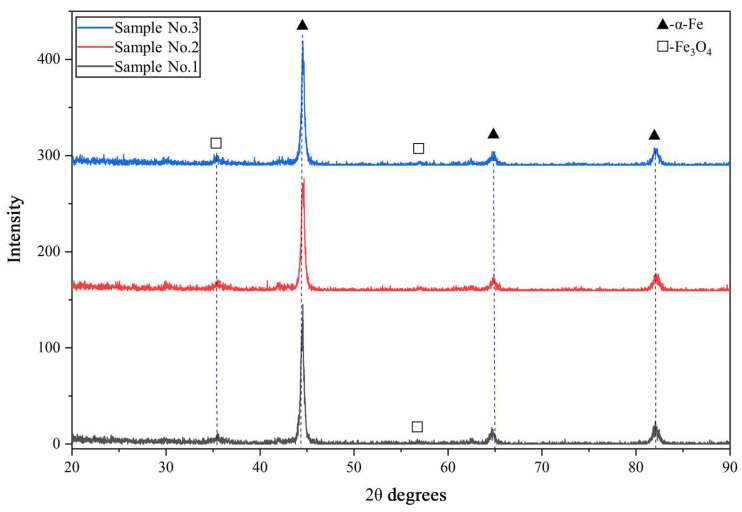
Diffraction patterns of coatings from 20Kh13 wire deposited at different air pressures (0.4, 0.5, and 0.6 MPa).

The results of X-ray phase analysis (Figure 5) confirmed the influence of the spraying mode on phase formation. In all investigated samples (0.4 MPa, 0.5 MPa, and 0.6 MPa), the main phase is α-Fe. It is interesting to note that, despite the equilibrium Fe–Cr–C diagram predicting the presence of carbides of type Me_3_C_6_ in 20Kh13 steels, carbide phases in the coating are practically absent. This is consistent with the microstructural observations and confirms that the high cooling rates prevent carbide precipitation from the solid solution. At the same time, increasing the air pressure leads to a noticeable increase in the amount of oxide phase Fe_3_O_4_ (magnetite). In sample №2, clear magnetite peaks appear, while in sample №3, their intensity becomes maximal. This correlates with the more active formation of oxide layers observed in the microstructure and can be explained by the increased oxygen content in the arc zone when the air flow is increased.

From a functional perspective, the presence of the Fe_3_O_4_ phase may play a dual role in the coating performance. On the one hand, magnetite can contribute to the formation of a compact tribo-oxide layer during sliding, which may stabilize friction behavior and reduce wear. On the other hand, excessive oxide content can act as brittle inclusions, potentially impairing coating cohesion and negatively affecting corrosion resistance if interconnected porosity is present.

**Figure 6:**
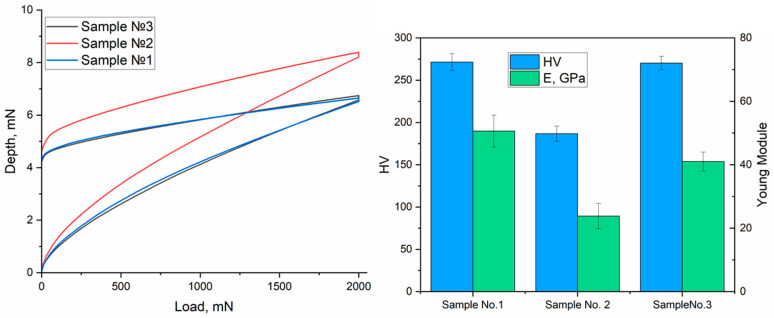
Dependence of indenter penetration depth on load and comparative diagram of microhardness (HV) and elastic modulus (E) of coatings obtained at different air pressures during electric arc spraying.

The results of the indentation depth measurements as a function of applied load are presented in Figure 6. The greatest indentation depth over the entire load range is exhibited by sample No. 2, obtained at an air pressure of 0.5 MPa. This indicates the lowest hardness and elastic modulus, which is consistent with the measurement results shown in the diagram (Figure 6). The reduced HV (186.7) and E (23.8 GPa) values are possibly due to the loose structure and lower layer density. Samples No. 1 and No. 3, obtained at air pressures of 0.4 and 0.6 MPa, respectively, show similar profiles on the graph. Their indentation depths are significantly smaller compared to sample No. 2 at the same loads. This indicates harder and denser coatings, which is confirmed by microhardness values (around 270 HV) and Young’s modulus (over 40 GPa). The slight differences in the curves between these two samples may be related to differences in morphology and layer thickness, but overall, both variants demonstrate high resistance to indentation.

**Figure 7:**
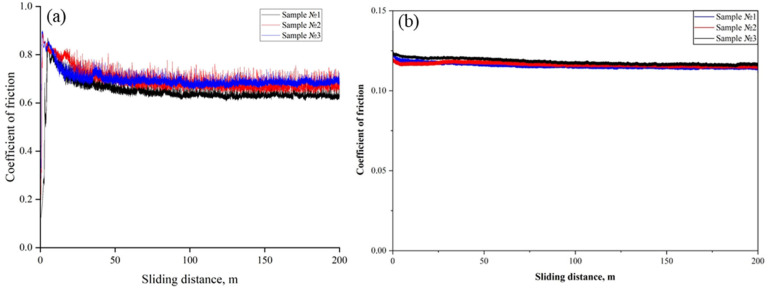
Friction coefficient as a function of sliding distance for coatings obtained at different air pressures: (**a**) under dry friction conditions, (**b**) in an oil-lubricated environment.

Tribological tests of the coatings produced by electric arc spraying at different air pressures (0.4–0.6 MPa), carried out in a ball-on-disk configuration under dry friction conditions, revealed differences in the coefficient of friction and its stability depending on the coating structure (Figure 7a). Sample No. 1 (0.4 MPa) had the lowest average friction coefficient—0.65—but also the greatest scatter of values (standard deviation 0.067), which is associated with the presence of porosity and unmelted inclusions in the coating. Sample No. 2 (0.5 MPa) showed the highest friction coefficient—0.70—with a standard deviation of 0.055, indicating a less dense and more heterogeneous structure. The best friction stability was demonstrated by sample No. 3 (0.6 MPa) with a friction coefficient of 0.68 and a minimum deviation—0.043—which is due to the dense and uniform structure of the coating with a minimal number of pores. Thus, despite having a slightly higher average coefficient of friction, the coating obtained at 0.6 MPa provides the most stable and predictable tribological characteristics under dry contact.

When oil was used as the lubricating medium (Figure 7b), a sharp decrease in the coefficient of friction was observed for all samples to values around 0.10–0.12. In all three cases, the friction-coefficient curve exhibits stable behavior without abrupt jumps, indicating good oil resistance and stable performance of the coatings under lubrication. A slight advantage in friction reduction is again shown by sample No. 3, confirming its high surface quality and denser structure, as previously revealed in microstructural and mechanical analyses.

**Figure 8:**
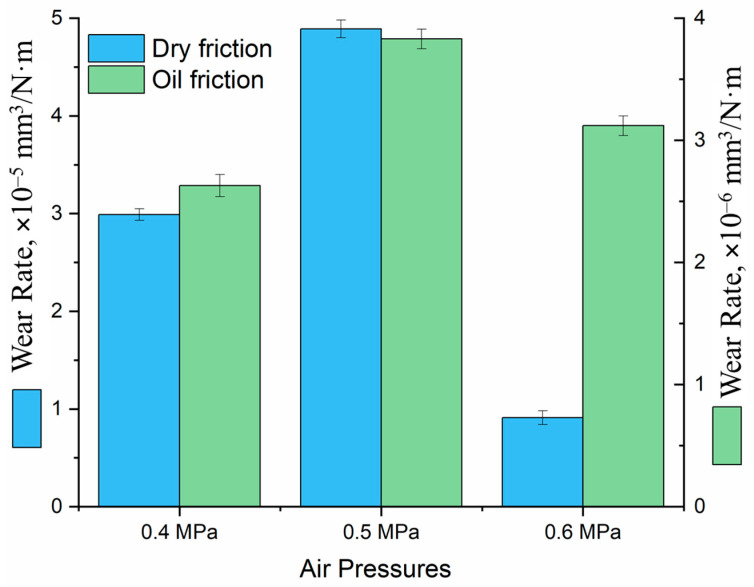
Wear rate of the coatings under dry and lubricated friction conditions.

Under dry friction conditions, the highest wear rate is observed for sample No. 2 (0.5 MPa) (Figure 8), which is attributed to its less dense and more heterogeneous coating structure. Sample No. 1 (0.4 MPa) exhibits a lower wear rate than sample No. 2 despite its higher porosity, whereas the lowest wear rate is demonstrated by sample No. 3 (0.6 MPa), characterized by the most compact and uniform microstructure. Under lubricated conditions, the wear rates of all coatings are significantly reduced compared to dry friction. The highest wear rate is still observed for sample No. 2 (0.5 MPa), which is attributed to its less dense and more heterogeneous structure. Sample No. 1 (0.4 MPa) exhibits a lower wear rate than sample No. 2, despite its higher porosity, which can be explained by lubricant retention within the pores and the formation of a more stable lubricating film. The lowest wear rate is demonstrated by sample No. 3 (0.6 MPa), indicating its superior wear resistance under lubrication.

**Figure 9:**
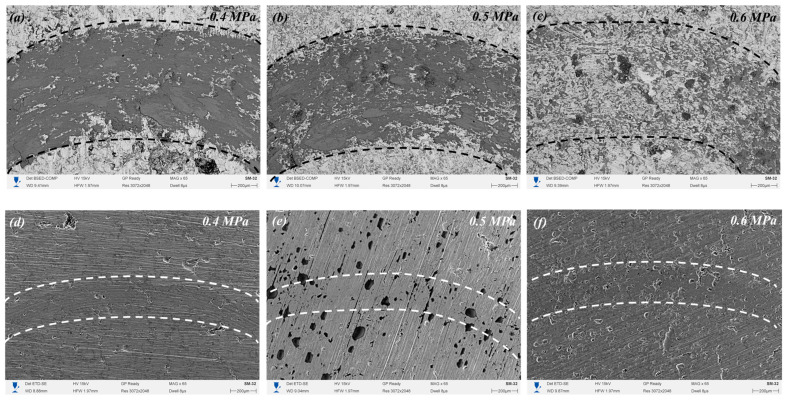
SEM images of the worn surfaces of coatings after tribological tests at different air pressures: (**a**–**c**) after dry friction at 0.4, 0.5, and 0.6 MPa, respectively; (**d**–**f**) after oil-lubricated friction at 0.4, 0.5, and 0.6 MPa, respectively.

As shown in Figure 9a–c, SEM images of the worn surfaces under dry friction conditions reveal clear differences in the wear mechanisms depending on the air pressure used during coating formation. At 0.4 MPa, the wear track shows a relatively smoother surface with some debris and signs of adhesive wear, which explains the lower COF but high variation due to surface irregularities and porosity. At 0.5 MPa, the wear surface is more damaged, with visible micro-cracks and delamination zones, indicating severe abrasive and fatigue wear, consistent with the highest COF and wear rate observed. At 0.6 MPa, the worn surface appears more uniform and compact, with signs of mild abrasive wear, confirming the high structural integrity and stability of the coating. As depicted in Figure 9d–f, under oil-lubricated conditions, all samples show significantly smoother wear tracks with minimal damage. Lubrication reduces direct contact and leads to the formation of protective films, especially effective in sample 3 (0.6 MPa), where the densest structure supports film stability.

## 4. Conclusions

The following conclusions can be drawn from the data obtained:

1. The electric arc spraying method using a two-layer scheme (30KhGSA interlayer and 20Kh13 top layer) ensures the formation of dense, adhesively strong coatings on 45 steel.
2. Increasing the atomizing air pressure to 0.6 MPa leads to a reduction in coating thickness but significantly improves its uniformity, reduces porosity (~2–3%), and stabilizes frictional characteristics.
3. The highest microhardness and elastic modulus were recorded at 0.4 MPa; however, the coatings produced at 0.6 MPa exhibit better structural integrity and minimal variability in mechanical and tribological properties.
4. All tested coatings demonstrated low friction coefficients in oil-lubricated conditions (~0.11) and stable behavior under dry friction. In addition, wear-rate analysis confirmed a clear dependence on the coating structure, with the lowest wear rate observed for the coating deposited at 0.6 MPa, indicating its superior wear resistance.
5. The obtained results confirm that the spraying regime at an air pressure of 0.6 MPa is optimal in terms of comprehensive performance, making it preferable for strengthening and restoring parts operating under heavy-duty conditions.

## Figures and Tables

**Table 1:** Chemical composition of steel 45 [25].

Steel Grade	C	Cu	Mn	As	Ni	P	S	Si	Fe
AISI 1045	0.47	0.28	0.78	0.07	0.20	0.035	0.040	0.27	97.85

**Table 2:** Electric arc spraying parameters for applying the top layer using 20Kh13 wire.

Sample	Voltage, V	Current, A	Time, s	Distance, mm	Pressure, MPa
Sample No. 1	60	130	25	200	0.4
Sample No. 2	60	130	25	200	0.5
Sample No. 3	60	130	25	200	0.6

## Data Availability

The original contributions presented in this study are included in the article. Further inquiries can be directed to the corresponding author.
